# Cossidae of the Socotra Archipelago (Yemen)

**DOI:** 10.3897/zookeys.122.1213

**Published:** 2011-08-11

**Authors:** Robert Borth, Povilas Ivinskis, Aidas Saldaitis, Roman Yakovlev

**Affiliations:** 1 LepBio, LLC; 2 Nature Research Centre, Akademijos 2, LT–08412 Vilnius-21, Lithuania; 3 Altai State University, Lenina 61, Barnaul, 656049, Russia

**Keywords:** Lepidoptera, Cossidae, *Meharia*, *Mormogystia*, *Aethalopteryx*, *Azygophleps*, new species, Socotra, Yemen

## Abstract

The faunistic composition of the family Cossidae (Lepidoptera) of the Socotra Archipelago is revised. Five species are recognized, including two new species (*Mormogystia brandstetteri* and *Meharia hackeri*), and dubious identifications and records are discussed. Adults and genitalia are illustrated and bionomic details, DNA barcodes and a synonymic checklist for Socotran cossids are provided. A review of their distribution reveals that at least 80 percent of Socotra’s cossids are unique to the archipelago, which is renowned for its endemism. A checklist listing all the species from generas *Meharia*, *Mormogystia*, *Aethalopteryx*, *Azygophleps*, as well as the synonymy and distribution is provided.

## Introduction

This paper results from a collaborative project “The Lepidoptera of Socotra Islands/Yemen – an integrative study of the fauna for reconstruction of evolutionary scenarios and for determination of conservation needs”, between the Zoologische Staatssammlung, (München, Germany), the Nature Research Centre (Vilnius, Lithuania) and Museum of Socotra Archipelago Conservation & Development Programme (Hadibo, Socotra, Yemen).

Socotra, which lies 240 km east of the Horn of Africa and 380 km south of the Arabian Peninsula, is a well-known source of material for biogeography and evolution studies – a living laboratory with a high degree of endemism. It was explored by both English (described by Hampson 1899) and Austrian (described by Rebel 1907) natural history expeditions just before the turn of the 20th century, but it remained effectively inaccessible during the 1900s due to its geographic isolation, extreme natural conditions and military concerns. Wranik’s 1999 summary of existing natural history knowledge addressed Lepidoptera conservation issues of the Socotra Archipelago, but was based primarily on information gained during those earlier expeditions. Collaboration of one of us (AS) with the SCDP, collecting from late February to early March and November 2008, March 2009 and January provided new data contributing to the understanding of Socotra’s Cossidae fauna.

The Socotra Archipelago consists of four islands with Socotra (130 kilometres in length and 30–40 kilometres in width) accounting for 95% of the archipelago’s land mass. Socotra, regarded as one of the most alien looking places on earth, has three main geographical features: (1) narrow coastal plains, (2) a limestone plateau extending across most of the island with karst caves, deep valleys and steep escarpments from 300 to 700 m, and (3) the Haghier Mountains in the centre of the island, which rise to 1,519 m (Miller and Cope 1996)

Socotra is a tropical desert with average highs between 27oC and 34oC and annual rainfall of only 130–170 mm. Rain is more intense in the higher mountains, which form the most important watershed and where many periodical watercourses run to the north and south. Permanent springs can also be found there, especially on the northern side. Otherwise, springs and streams are sporadic relying on rainfall. Climate conditions, rainfalls and major wind systems are dominated by seasonal monsoons of the Indian Ocean with most rain occurring during the Northern Hemisphere winter. The monsoon season causes strong winds and high seas, which cut off the island completely during the time of the southwest monsoon from May to September (Miller and Cope 1996, Wranik 1999).

The Socotra Archipelago is thought to have been part of the Gondwana supercontinent before it detached during the Miocene. In Tertiary times, Socotra was separated as part of a fault block from the African-Arabic tectonic plate and was formed coincident with the Gulf of Aden. As a result of its extremely long isolation, Socotra is of major biogeographical interest and more than one third of all its plants and possibly animals are found nowhere else. Botanists rank Socotra’s flora, including the extraordinary dragon´s blood tree *Dracaena cinnabari*,to be among the most important and endangered island floras of the world. It is generally suggested that the endemic plants and animals are relicts and descendants of ancient flora and fauna, which have survived since the Mesozoic era (Miller and Cope 1996, Wranik 1999).

Socotra Archipelago fauna is composed of tropical-subtropical arboreal and eremic elements derived from African, Asian or south-Arabian and endemic origins (Wranik 1999). No comprehensive investigations of the insular fauna of Socotra are available and ambiguous taxonomic definitions have repressed faunistic analysis and development of species checklists. Wranik (1999) examined a limited number of groups including Odonata in which only one of 20 species is endemic and Saltatoria where over half of about 50 species are endemic. Wranik found Tenebrionidae to have the highest level of endemism: most of about 30 species are endemic, indicating a Somalarabic relationship, while others are may be relicts from a more ancient fauna already extinct on the mainland.

About 250 species of Lepidoptera are currently reported from Socotra in the literature including 30 species of Rhopalocera(Hacker 1999), 89 Noctuidae (Hacker and Saldaitis 2010), over 40 Pyralidae(Hacker 1999), 28 Geometridae (Hausmann 2009) and others from less studied groups. Rebel (1907) suggested that 1/3 of the Lepidoptera fauna of the Socotra Archipelago was endemic, with a dominance of Afrotropical relationships.

We present five Cossidae species from Socotra, excluding *Eremocossus proleuca* (Hampson, 1896) and *Azygophleps inclusa* (Walker, 1856) which were mistakenly attributed to Socotra by Hampson (1903), Rebel (1907) and Hacker (1999). *Eremocossus proleuca* which was erroneously synonymized by Wiltshire (1980) as *Eremocossus reibellii* (Oberthür, 1876) does not occur on Socotra but was probably confused with one of the two new Cossidae species described in this paper. *Azygophleps inclusa* is distributed only in tropical Africa and differs from the similar *Azygophleps larseni* which is distributed in the Arabian peninsula and Socotra island.

## Materials and methods

Material was collected in February through early March and November 2008, March 2009 and January 2010 using artificial light.

DNA barcodes (658 base pairs of Cytochrome Oxidase Subunit I 5’ region, (COI-5P) were sequenced by Paul Hebert’s laboratory at the University of Guelph for 15 Cossidae specimens.

### Abbreviations

LT	locus typus (type locality)

### Abbreviations of depositories

ASV	private collection of Aidas Saldaitis (Vilnius, Lithuania)

BMNH	Natural History Museum (London, UK)

JBW	private collection of Johann Brandstetter (Winhöring/Kronberg, Germany)

LLE	private collection of Lutz Lehmann (Eisenhüttenstadt, Germany)

MNHN	Muséum National d’Histoire Naturelle (Paris, France)

MWM/ZSM	Museum Thomas Witt (Munich, Germany)/Zoologische Staatssammlung, München (Germany)

NRCV	Nature Research Centre (Vilnius, Lithuania)

RYB	private collection of Roman Yakovlev (Barnaul, Russia)

SCDP	Museum of Socotra Archipelago Conservation & Development Programme

## Systematic accounts

### Genus Mormogystia

Schoorl, 1990

http://species-id.net/wiki/Mormogystia

Mormogystia  Schoorl, 1990, Zool. Verhandelingen 263: 75–78. Type species – *Cossus reibellii* Oberthür, 1876.

#### Diagnosis.

 *Mormogystia* is distinguished from all other Cossidae genus by having large silvery areas on the forewing.

#### Description.

 Medium sized, brightly coloured moths. Male antennae bipectinate with very short processes; female antennal pecten much reduced. Large silvery areas on the forewing forming fasciae make this the only Cossidae genus to have such a high contrast pattern. Hindwings are uniform.

**Male genitalia.** Uncus elongate, with tapering or rounded broad apex; arms of gnathos short, fused to form a medium-size gnathos densely covered with small spines; valvae shovel-shaped, with pronounced sacculus and a large triangular costal projection; transtilla projections short, thick and uncinate; juxta saddle-shaped, with long lateral projections directed upwards; saccus massive, semicircular; aedeagus short, straight, thick; vesica opening located dorsoapically, its edges with short, spiny processes; vesica without cornutus.

**Female genitalia**. Short oviductus; papillae anales wide, elliptic; apophyses posteriores ⅓ longer than apophyses anteriores; ostium broad, covered with falciform postvaginal plate; ductus wide, sclerotised; bursa membranous, sack-shaped, without signa.

#### Remarks.

This small genus includes four species distributed in north Africa, Levante, Arabian peninsula and Kenya (Yakovlev 2011 ).

### Mormogystia brandstetteri

Saldaitis, Ivinskis & Yakovlev sp. n.

urn:lsid:zoobank.org:act:48E8D1AE-EAD6-4DBD-AA0A-AC40BB375524

http://species-id.net/wiki/Mormogystia_brandstetteri

Figs 1, 2 21 27 

#### Type material.

**Figures 1–6.**
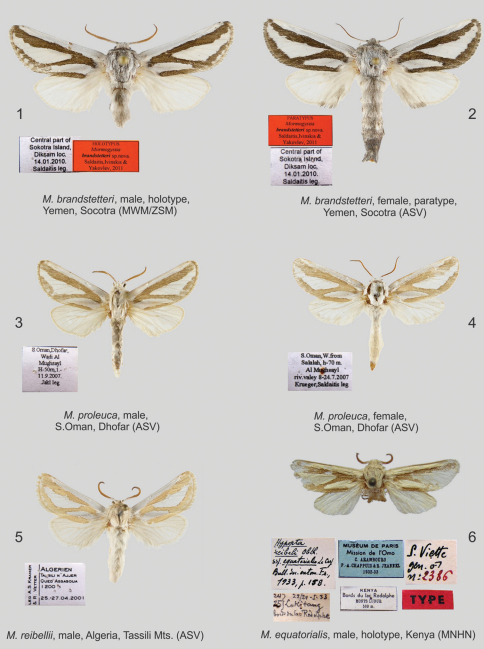
*Mormogystia* spp., adults. **1** *Mormogystia brandstetteri*, male, holotype, Yemen, Socotra (MWM/ZSM) **2** *Mormogystia brandstetteri*, female, paratype, Yemen, Socotra (ASV) **3** *Mormogystia proleuca*, male, S.Oman, Dhofar (ASV) **4** *Mormogystia proleuca*, female, S.Oman, Dhofar (ASV) **5** *Mormogystia reibellii*, male, Algeria, Tassili Mts. (ASV) **6** *Mormogystia equatorialis*, male, holotype, Kenya (MNHN);

 **Holotype** ♂ (Fig. 1), central part of Socotra Island, Diksam loc., 14 January 2010, leg. A. Saldaitis (deposited in MWM/ZSM; slide No. BJ 1524). **Paratypes**: 77 ♂ and ♀ (Fig. 2), with same labels as holotype; Socotra Archipelago, Samha Island W., N 12°09', E 052°59', 23–24 February 2008, leg. A. Saldaitis; Socotra Archipelago, Abd al Kuri Island, Towanie vill. env., N 12°10', E 052°13', 25–27 February 2008, leg. A. Saldaitis; Socotra Island, Di Hamri loc., 1 March 2008, leg. Saldaitis; Socotra Island, Di Hamri loc., 20–21 November 2008, leg. Saldaitiene & Saldaitis; Socotra Island, hills near Hadibu, 21 March 2009, leg. A. Saldaitis; Socotra Island, Diksam canyon, 23 March 2009, leg. A. Saldaitis; W Socotra, Shuab, coast line, mangroves, 24 March 2009, leg. A. Saldaitis; N Socotra Island, Ayhft valley, 22 November 2008, leg. A. Saldaitis; S Socotra Island, Wadi Difarroha South side, 15 January 2010, leg. A. Saldaitis; N Socotra Island, Ayhft valley, 12 January 2010, leg. A. Saldaitis; N Socotra Island, Wadi Kam, 13 January 2010, leg. A. Saldaitis; N Socotra Island, top of Ayhft valley, 17 January 2010, leg. A. Saldaitis; E Socotra Island, sand dunes near Irisseyl loc., 18 January 2010, leg. A. Saldaitis; NE Socotra Island, Wadi Difarroha, North side, 19 January 2010, leg. A. Saldaitis (coll. ASV; JBW; LLE; MWM/ZSM; NRCV; RYB). Slide No. BJ 1532 (female).

#### Diagnosis.

The new species differs from the related species *Mormogystia reibellii* (Oberthür, 1876) (Fig. 5), *Mormogystia proleuca* (Hampson in Walsingham et Hampson, 1896) (Figs 3, 4) and *Mormogystia equatorialis* (Le Cerf 1933) (Fig. 6) in external appearance, genitalia, DNA and distribution. The new species has a larger wingspan than its congeners: *Mormogystia brandstetteri* sp. n. 33–35 mm, *Mormogystia reibellii* 27–31 mm, *Mormogystia proleuca* 25–29 mm, *Mormogystia equatorialis* 26 mm. All species of the genus *Mormogystia* have a similar forewing pattern, but the ground colour of the new species is black as opposed to light ochre, light brown and brown, respectively, for *Mormogystia reibellii*, *Mormogystia proleuca* and *Mormogystia equatorialis*. The head, thorax and abdomen of *Mormogystia brandstetteri* are intense grey compared to light yellow in *Mormogystia reibellii* andlight yellow and white in *Mormogystia proleuca* and *Mormogystia equatorialis*. Unlike the other species *Mormogystia brandstetteri* has a black costal spot on the ventral hindwing; in *Mormogystia reibellii* (Figs 23, 24)and *Mormogystia proleuca* (Fig. 22) uncus apically tapering, strongly sclerotised valvae not widening and not forming a straight angle; in *Mormogystia brandstetteri* uncus broad, apex of valvae form a straight angle; in *Mormogystia reibellii* and *Mormogystia proleuca* saccus rounded, apically without denticle, whereas in the new species saccus pointed, apically with a denticle; *Mormogystia proleuca* aedeagus at the basal end markedly narrowing, gradually widening towards apex; *Mormogystia reibellii* aedeagus of the same width from its middle to apex; in *Mormogystia brandstetteri* aedeagus widening at proximal end, gradually tapering towards apex. In the most closely related species, *Mormogystia proleuca*, the bursa is apically broader than basally (Fig. 28) unlike in the new species where the corpus bursae is significantly broader.

#### Distribution.

 *Mormogystia brandstetteri* is endemic to the Socotra Archipelago while *Mormogystia reibellii* is distributed in North Africa and the northern part of the Arabian peninsula, *Mormogystia proleuca* is found in the southern part of the peninsula, and *Mormogystia equatorialis* is widespread in Kenya. Hampson (1903) and Rebel (1907) believed *Mormogystia proleuca* to be endemic to the Socotra Archipelago and later Hacker (1999) reported *Mormogystia reibellii* from Socotra, but the new species described herein was probably implied.

#### Molecular Analysis.

 While molecular results alone are insufficient to definitively separate *Mormogystia brandstetteri* from *Mormogystia proleuca*, they help corroborate the morphological evidence. Evolutionary distances using the Kimura two-parameter model for comparing four specimens of *Mormogystia brandstetteri* to four *Mormogystia proleuca* and to three *Mormogystia reibellii* specimens, was at least 1.55% and 5.65%, respectively.

#### Description.

 **Male**: Forewing costal margin length of holotype 15 mm, wingspan 33 mm; mean forewing length of paratypes 16 mm, wingspan 35 mm; head, thorax, abdomen and tegulae grey; antennae bipectinate, ½ the length of forewing; ground colour of forewing black, with white silvery pattern. Three white silvery patches form the pattern: fascia of even width runs along the entire costal margin, median fascia widening medially reaches the outer margin of forewing; lower silver patch originates at basal edge and extends along dorsal wing margin to middle. This patch enclosed by ground colour; adterminal line white; fringe grey. Dorsal surface of forewing greyish-white; costal, outer and dorsal margins greyish-black. Hindwing uniform, white, with greyish black spot at costal margin. **Female** (Fig. 2): Forewing length of allotype 23 mm, wingspan 48 mm; antennae filiform; wing pattern as in males. **Intraspecific variation.** Adterminal line in some specimens missing; contours of silvery spots forming the pattern vary; hindwings grey.

**Male genitalia** (Fig. 21). Uncus broad, slightly narrower than its length; apex wide, slightly rounded; arms of gnathos long and strong; gnathos very broad, with rounded apex; apex of saccus gradually tapering, with a pointed denticle; valvae symmetrical, with straight margins, gradually widening apically; costal margin with strong and wide sclerotisation; apex flat; arms of transtilla medium sized, strong, denticle-shaped; juxta large, strongly sclerotised, belt-shaped with a small indentation apically and a conspicuous boat-shaped margin at the basal area; aedeagus strong, straight, large, widening at the proximal end; vesica simple, wide, without cornuti.

**Female genitalia** (Fig. 28). Papilla analis narrow, covered with short, thin setae; apophysis posterioris 1.4 times longer than apophysis anterioris; antevaginal plate belt-shaped, pointed at the ends; ductus bursae sclerotised; corpus bursae shaped like a long narrow sac, not sclerotised; signa absent.

#### Bionomics and distribution.

 Both males and females of the new species were strongly attracted to light and were distributed in almost all habitats of Socotra Island as well as the smaller islands of the archipelago – Samha and Abd al Kuri. *Acacia* is a likely food plant for *Mormogystia brandstetteri* as larvae of the closely allied species *Mormogystia proleuca* feed on *Acacia* [Hampson, 1896]. Also, the new species is especially abundant in the central part of the island, in deeper canyons or rich oasis-like valleys where forests haven’t been cut for fuel like elsewhere on the island. Diksam canyon (Fig. 30), a prime locality for *Mormogystia brandstetteri*, contains the following plants: *Acacia pennivenia*, *Jatropha unicostata*, *Lycium socotranum*, *Gnidia socotrana*, *Buxus hildebrandtii*, *Croton socotranus*, *Leucas virgata*, *Cissus hamaderohensis*, *Punica protopunica*, *Ficus vasta*, *Euphorbia socotrana*, *Jathropha unicostata*, *Lycium socotranum*, *Gnidia socotrana*, *Buxus hildebrandtii*, *Trichocalyx* sp., *Mitolepis intricata*, *Ballochia* spp., *Aloe perryi*, *Adenium obesum*, *Asparagus africanus*, *Seddera fastigiata*, *Aerva lanata*, *Rhinacanthus scoparius*, *Levandula nimmoi*, *Ocimum forskahlei*, *Cissus hamaderohensis* (Miller and Cope 1996). *Mormogystia brandstetteri* flies with several other Socotra Archipelago endemic moths such as *Meharia yakovlevi* Saldaitis & Ivinskis, 2010, *Aethalopteryx diksami* Yakovlev & Saldaitis, 2010, (Cossidae), *Pelosia sokotrensis* (Hampson, 1900), *Siccia butvilai* Saldaitis & Ivinskis, 2008, (Arctiidae), *Cerocala socotrensis* Hampson, 1899, *Agrotis brachypecten* Hampson, 1899, *Leucania diopsis* Hampson, 1905 and *Mythimna sokotrensis* Hreblay, 1996 (Noctuidae).

#### Etymology.

 The new species is dedicated to our good friend Johann Brandstetter, an eminent German painter and entomologist.

### Genus Meharia

Chrétien, 1915

http://species-id.net/wiki/Meharia

Meharia  Chrétien, 1915, Ann. Soc. Ent. Fr. 84: 367. Type – species: *Meharia incurvariella* Chrétien, 1915.
Blalia  Rungs, 1943; Rungs, [1943], 1942, Bull. Soc. Sc. Nat. Maroc. 22: 174. Type species – *Blalia vittata* Rungs, [1943]. [Synonymy]

#### Diagnosis.

*Meharia* is distinguished from all other Cossidae genus by a number of apomorphous characters: the specific “tineoid appearance”, the reduction of the lateral processes of the juxta, the specific dorsolateral sclerotization of the asymmetric aedeagus and the specific ribbon – like epiphysis.

#### Description.

 These are small to medium sized moths, females larger; eyes naked; male and female antennae bipectinate along their length; proboscis reduced; legs long, slender; foretibia bearing a ribbon-like epiphysis; forewing elongate, rounded on the outer margin; forewing pattern has alternate dark and pale spots and bands transversely; hindwing uniform.

**Male genitalia**. Simple; uncus unpaired, short, beak-shaped; tegumen massive; arms of gnathos short, slightly broadened distally, fused to form small gnathos; valvae short, broad, with no harpe and processes costally; juxta without lateral processes, simple; saccus protruding backwards, small; aedeagus rather long, slightly curved and asymmetical due to dorsoapical sclerotisation.

**Female genitalia**. Ovipositor lobes short, slightly acute apically, covered with relatively short, thick bristles, in the shape of triangular sclerites, with long and rather wide apophyses posteriores on the lower part, strongly widening oar-like in the cranial fourth and bearing a slender membranous-like border; tergite and sternite of the 8th segment fused to form a complete circle; sternite slightly swollen, membranous caudally; tergite strongly elongate, bearing a pair of apophyses anteriores, widening oar-like cranially, approximately as long as ½ the length of apophyses posteriores; opening of ostium strongly protruding cranially, located on membrane between the 7th and 8th segments; ostium membranous, with poorly sclerotized lateral bands; antrum membranous, tube-shaped, 1½ times longer than the 8th tergite, narrowing sharply, separate form membranous ductus bursae; corpus bursae membranous, saccular, without signa.

#### Remarks.

 Eleven species of *Meharia* have been reported so far (Yakovlev and Saldaitis 2008), primarily from the deserts and arid mountains of the Western Palearctic and Africa.

### Meharia hackeri

Saldaitis, Ivinskis & Yakovlev sp. n.

urn:lsid:zoobank.org:act:730024CA-3646-4660-9FBD-C307773D0E94

http://species-id.net/wiki/Meharia_hackeri

Figs 9, 10, 13, 14 

#### Type material.

 **Holotype** ♀ (Fig. 9), NE Socotra Island, Wadi Difarroha, North side, 19 January 2010. leg. A. Saldaitis (deposited in MWM/ZSM); (slide No PI 2011/1) **Paratypes**: 3 ♀ (Fig. 10), S Socotra Island, Wadi Difarroha, South side, 15 January 2010. leg. A. Saldaitis; (coll. ASV; MWM/ZSM); (slide No BJ 1523).

#### Diagnosis.

The new species differs from the related species *Meharia acuta* Wiltshire, 1982 (Figs 8, 12) by forewing pattern, DNA and distribution. In *Meharia acuta*, the basal spot at the costal wing margin is missing. *Meharia hackeri* has a straight basal fascia at the costal wing margin for ¼ the length of forewing and a narrow white fascia, with a wide interruption antemedially and a narrow interruption tornally, running along the entire inner margin. *Meharia acuta* has no such fascia, but has a wide subterminal band. DNA barcodes clearly separate *Meharia hackeri* from *Meharia acuta*. Three identical sequences of *Meharia hackeri* were compared to those of a single *Meharia acuta* specimen resulting in a significant 7.48% variation.

*Meharia acuta* is distributed in the Arabic peninsula, *Meharia hackeri* is endemic to Socotra Island.

#### Description.

 **Female**: Forewing costal margin length of holotype 10 mm, wingspan 21 mm; forewing length of paratypes 11 mm, wingspan 22 mm; antennae slightly longer than half the length of forewing; bipectinate, color white, black at base; head and tegular yellowish-white; labial palpi yellowish brown, white at base; ground colour of forewings yellowish-brown with white longitudinal fascia forming wing pattern, basal fascia in the costal area straight, running to ¼ the length of forewing, curved fascia extending medially from inner margin to ⅔ the length of forewing, its extension ends at terminal wing margin, medially the fascia and its interrupted portion in terminal area bordered by dark brown scales with black inserts; narrow white fascia, widely interrupted antemedially and narrowly interrupted tornally, runs along the entire inner margin, cilia yellowish-white, ventral forewing brown; hindwing greyish-yellow, cilia light brown, ventral hindwing brown.

**Male genitalia:** unknown.

**Female genitalia** (Figs 13, 14): Papilla analis triangular, covered with short, thin, very long setae; apophysis posterioris about the same length as papilla analis; apophysis anterioris very short, broad, with V-shaped sclerotisation apically; ostium concave; antrum weakly sclerotised basally with a loop forming very narrow ductus bursae; corpus bursae not sclerotised, shaped like a small sac.

#### Bionomics and distribution.

 Known only from the central part of Socotra Island. *Meharia hackeri* is likely endemic to Socotra Island. All specimens were collected in mid-January; *Meharia hackeri* females were attracted to light and appear to have a very local distribution as the species was discovered only in Difarroha Valley (Fig. 31). The new species was collected in the central part of the country in an oasis-type valley dominated by various tree and shrub species such as: *Jatropha unicostata*, *Lycium socotranum*, *Gnidia socotrana*, *Buxus hildebrandtii*, *Croton socotranus*, *Punica protopunica*, *Ficus vasta*, *Euphorbia socotrana*, *Jathropha unicostata*, *Mitolepis intricata*, *Aloe perryi*, *Adenium obesum* (Miller and Cope 1996). It flies with several other Socotra Archipelago endemic moths such as *Meharia yakovlevi* Saldaitis & Ivinskis, 2010, (Cossidae), *Pelosia sokotrensis* (Hampson, 1900), (Arctiidae),*Cerocala socotrensis* Hampson, 1899, *Agrotis brachypecten* Hampson, 1899, *Plecoptera butkevicii* Hacker & Saldaitis, 2010, *Acantholipes canofusca* Hacker & Saldaitis, 2010, *Stenosticta wiltshirei* Hacker, Saldaitis & Ivinskis, 2010 (Noctuidae).

#### Etymology.

 The new species name is dedicated to Hermann Hacker, a prominent German lepidopterist, who has contributed much to the investigation of macro-moths of the Arabian peninsula and Africa.

### Meharia yakovlevi

Saldaitis & Ivinskis, 2010

http://species-id.net/wiki/Meharia_yakovlevi

Figs 7, 11

Meharia yakovlevi  Saldaitis & Ivinskis, 2010a, Esperiana 15: 379.

#### Description.

 **Male genitalia** (Fig. 11): The authors examined the genital structures of several more male specimens, noting that the valvae are variable in shape being slightly narrower and slightly concave in costal and dorsal areas. The vesica is narrow, long, and almost the same length as aedeagus.

#### Distribution.

**Figures 7–14.**
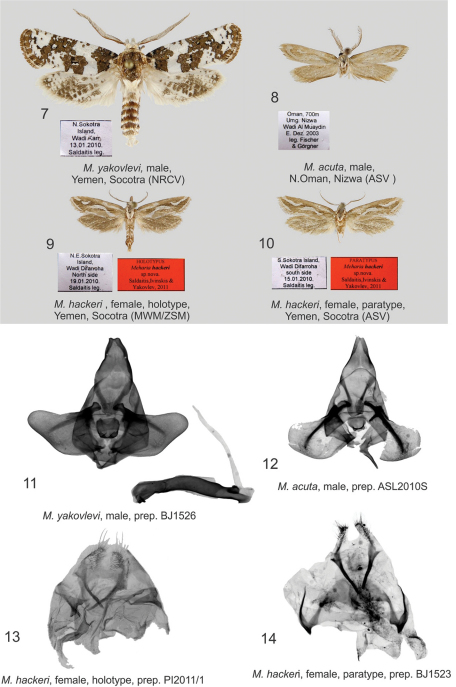
*Meharia* spp., adults and genitalia. **7** *Meharia yakovlevi*, male, Yemen, Socotra (NRCV) **8** *Meharia acuta*, male, N.Oman, Nizwa (ASV ) **9** *Meharia hackeri* , female, holotype, Yemen, Socotra (MWM/ZSM) **10** *Meharia hackeri*, female, paratype, Yemen, Socotra (ASV) **11** *Meharia yakovlevi*, male, prep. BJ1526 **12** *Meharia acuta*, male, prep. ASL2010S **13** *Meharia hackeri*, female, holotype, prep. PI2011/1 **14** *Meharia hackeri*, female, paratype, prep. BJ1523;

 This species was described from a single male. This specimen was collected in Hadibu environs, in the hills covered by dense shrubby vegetation dominated by the following plants: *Rhus thyrsiflora*, *Buxus hildebrandtii*, *Carphalea obovata*, *Sterculia africana*, *Dracaena cinnabari*, *Rhus thyrsiflora*, *Carphalea obovata*, *Tamarindus indica*, *Commiphora socotrana*, *Commiphora ornifolia*, *Commiphora parvifolia*, *Boswellia ameero*, *Boswellia elongata*, *Boswellia bullata*, *Boswellia dioscorides*, *Boswellia nana*, *Punica protopunica*, *Acacia pennivenia*, *Cephalocroton socotranus*, *Indigofera socotrana*, *Dirachma socotrana*, *Allophylus rubifolius*, *Maerua socotrana*, *Acridocarpus socotranus*, *Sterculia africana*, *Zizyphus spina–christi*, *Ficus vasta*, *Ficus salicifolia*, *Arthrocarpum gracile*, *Ormocarpum caeruleum* (Miller and Cope 1996). In January 2010, five more specimens were collected (1 ♂, N Socotra Island, Wadi Kam, 13 January 2010, leg. A. Saldaitis (Fig. 7); 2 ♂ central part of Socotra Island, Diksam loc., 14 January 2010, leg. A. Saldaitis; 2 ♂ S Socotra Island, Wadi Difarroha South side, 15 January 2010, leg. A. Saldaitis). *Meharia yakovlevi* appears to be a very rare and local species showing a slightly higher abundance in the central part of the country with oasis-like valleys and canyons with relict woody vegetation. These habitats were dominated by the following plants: *Jatropha unicostata*, *Lycium socotranum*, *Gnidia socotrana*, *Buxus hildebrandtii*, *Croton socotranus*, *Punica protopunica*, *Ficus vasta*, *Euphorbia socotrana*, *Jathropha unicostata*, *Mitolepis intricata*, *Aloe perryi*, *Adenium obesum* (Miller and Cope 1996).

### Genus Aethalopteryx

Schoorl, 1990

http://species-id.net/wiki/Aethalopteryx

Schoorl, 1990, Zool. Verhandelingen 263: 174–175. Type species – *Phragmatoecia atrireta* Hampson, 1910.

#### Diagnosis.

*Aethalopteryx* is distinguished from close *Trismelasmos* Schoorl, 1990, *Acosma* Yakovlev, 2011, *Strigocossus* Houlbert, 1916 and *Azygophleps*Hampson**,** 1892 genus by having cup-shaped antennae in both sexes, forewings with slight reticulated patterns and reduced arms in males gnathos and particularly genital structure of the females.

#### Description.

 Medium sized moths. Male and female antennae cup-shaped; forewing elongate with slight reticular pattern, often with a spot in the costal area and spots in the postdiscal area; hindwing with indistinct reticular pattern.

**Male genitalia.** Uncus long, thin, basally considerably narrower than width of tegumen; arms of gnathos reduced; tegumen massive; valvae with slightly uneven margins and with rounded apex; saccus massive, semicircular; juxta broad, with wide leaf-shaped lateral processes; aedeagus slightly bent, vesica with a long belt-shaped sclerite forming the projection of lateral aedeagus wall.

**Female genitalia.** Form short oviductus; papilla analis elongate, gradually narrowing; apophyses posteriores twice the length of apophyses anteriores which are furcate at basal part; ductus membranous, broad, very short; corpus bursae shaped like a long narrow sac, with a star-shaped signum on the lateral surface; bulla located in basal third of bursa on a long membranous ductus.

#### Remarks.

 Thirty-four species of *Aethalopteryx* have been reported (Yakovlev 2011), primarily from the east Africa with some distributed elsewhere in Africa or in the Arabian peninsula.

### Aethalopteryx diksami

Yakovlev & Saldaitis, 2010

http://species-id.net/wiki/Aethalopteryx_diksami

Figs 20 26

Aethalopteryx diksami  Yakovlev & Saldaitis, 2010, Esperiana Memoir 5: 334, Pl. 20: fig. 5.

#### Description. 

**Male genitalia** (Fig. 26). The authors examined several more male specimens and found some variation in the genital structures. Valvae of newly examined specimens were significantly wider; apex rounded; costal margin even; vertical juxta processes not tapering, with obtuse apices; vesica simple, long, tapering, almost the same length as aedeagus.

#### Distribution.

 A newly described species, highly local, known only from the central part of Socotra Island from two valleys: the Diksam canyon (Fig. 30) and the Difarroha valley (Fig. 31), which are characterized by the following relict woody vegetation: *Dracaena cinnabari*, *Buxus hildebrandtii*, *Croton socotranus* and numerous other endemic plants (Miller and Cope 1996).

### Genus Azygophleps

Hampson, 1892

http://species-id.net/wiki/Azygophleps

Azygophleps  Hampson, 1892, Fauna Brit. India **1**: 309.Type species – *Hepialis scalaris* Fabricius, 1775.
Azygophlebs  Aurivillius, 1925, Ergeb. Zweit. Deutsch. Zentral-Afrika-Exped. 1910–1911: 1349; An incorrect subsequent spelling of *Azygophleps* Hampson, 1892. [Synonymy]

#### Diagnosis.

*Azygophleps* is distinguished from similar genera such as *Sansara* Yakovlev, 2004, *Strigocossus* Houlbert, 1916 and *Aethalopteryx* Schoorl, 1990 by its females’ apically bipectinate antennae, its long forewings rounded at the apex, the abscence of arms in its males’ gnathos, its thick aedeagus, and a short, wide ductus and corpus with a small star-like signum in its females.

#### Description.

 Medium sized moths. Male antennae cup-shaped, those of female bipectinate (apically with gradually reducing pectin); forewing long, with rounded apex, with dense reticular pattern formed by transverse lines and spots; hindwing lightly coloured and uniform.

**Male genitalia**. Uncus medium-sized, apically hooked; arms of gnathos absent; tegumen medium sized, usually wider than basal part of uncus; valvae with almost straight margins and wide rounded apex; juxta medium-sized, with long, narrow, well-sclerotised lateral processes; saccus semicircular, massive; aedeagus thick, with long sclera forming aedeagus wall.

**Female genitalia**. Forming long ovipositor; papilla analis stretched, slightly tapering towards apex; apophyses posteriores more than twice as long as apophyses anteriores which are forked basally; ductus short, wide, sclerotised at base; corpus sac-shaped, with a small star-like signum; bulla located on the apical part of bursa.

#### Remarks.

 Twenty-eight species of *Azygophleps* have been reported (Yakovlev 2011), primarily throughout Africa with a few species distributed in the Arabian peninsula and Asia.

**Figures 15–20.**
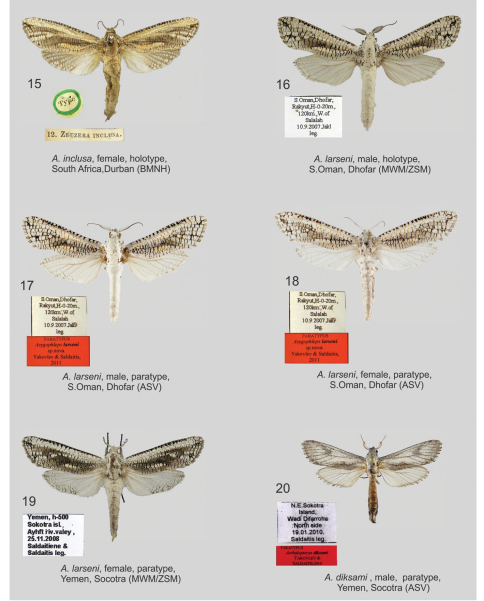
*Azygophleps* spp. and *Aethalopteryx* spp., adults. **15** *Azygophleps inclusa*, female, holotype, South Africa, Durban (BMNH) **16** *Azygophleps larseni*, male, holotype, S.Oman, Dhofar (MWM/ZSM) **17** *Azygophleps larseni*, male, paratype, S.Oman, Dhofar (ASV) **18** *Azygophleps larseni*, female, paratype, S.Oman, Dhofar (ASV) **19** *Azygophleps larseni*, female, paratype, Yemen, Socotra (MWM/ZSM) **20** *Aethalopteryx diksami*, male, paratype, Yemen, Socotra (ASV);

**Figures 21–24.**
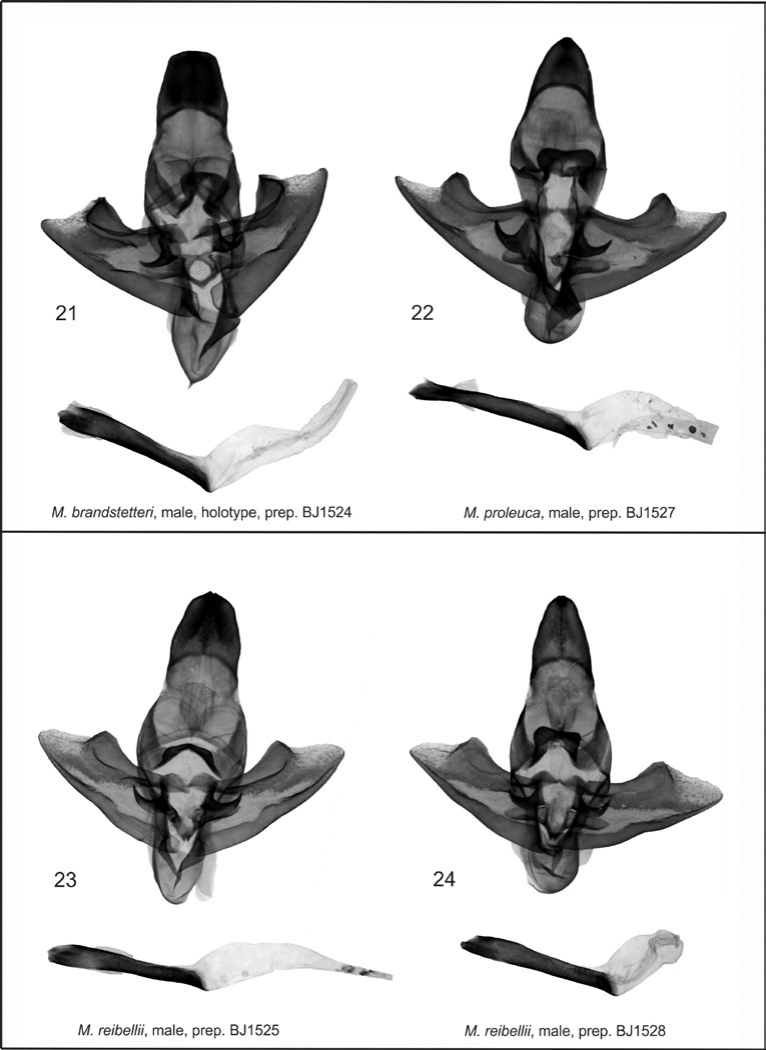
*Mormogystia* spp.,males genitalia. **21** *Mormogystia brandstetteri*, male, holotype, prep. BJ1524 **22** *Mormogystia proleuca*, male, prep. BJ1527 **23** *Mormogystia reibellii*, male, prep. BJ1525 **24** *Mormogystia reibellii*, male, prep. BJ1528;

**Figures 25–29.**
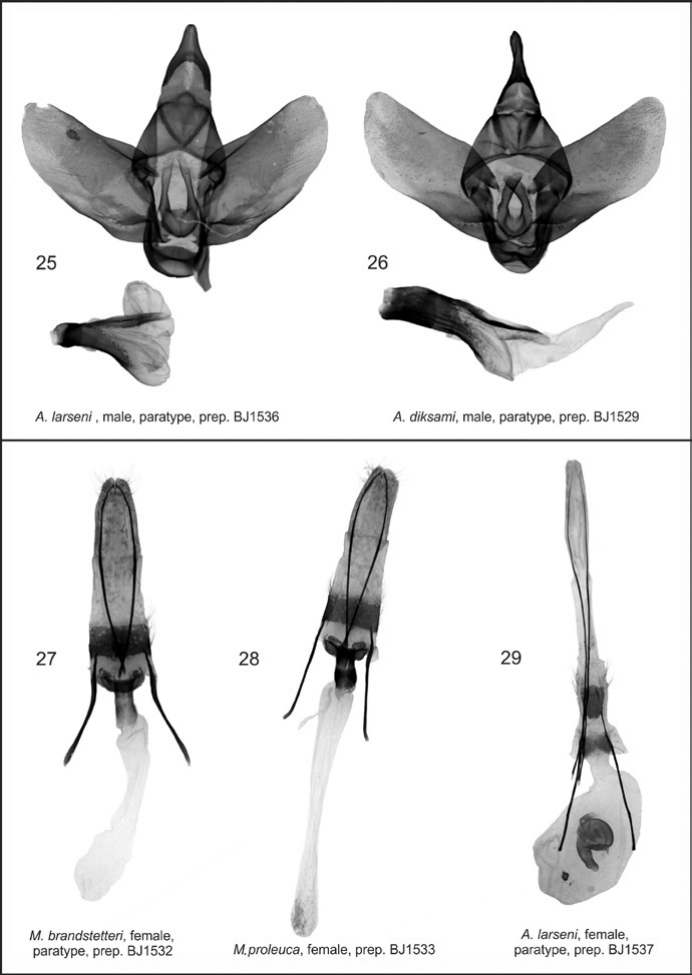
*Azygophleps* sp. and *Aethalopteryx* sp., male genitalia and *Mormogystia* spp. and *Azygophleps* sp., female genitalia. **25** *Azygophleps larseni*, male, paratype, prep. BJ1536 **26** *Aethalopteryx diksami*, male, paratype, prep. BJ1529 **27** *Mormogystia brandstetteri*, female, paratype, prep. BJ1532; 28. *Mormogystia proleuca*, female, prep. BJ1533; 29. *Azygophleps larseni*, female, paratype, prep. BJ1537;

### Azygophleps larseni

Yakovlev & Saldaitis, 2011

http://species-id.net/wiki/Azygophleps_larseni

Figs 16–19 25, 29

Azygophleps larseni  Yakovlev & Saldaitis, 2011, Neue Entomologische Nachrichten 66: 84, Pl. 8: Figs 28–29.

#### Description.

 Female size and wing pattern similar to the male, however in Socotra specimens the pattern of the forewing is darker and the dorsal margin of hindwing has a reticular pattern. Antennae in females are cup-shaped as in males, but pecten are significantly shorter. Both female specimens from Socotra Island differ from typical *Azygophleps larseni* from Oman in external appearance. Without opportunity to compare *Azygophleps larseni* male genitalia we abstained from assigning the Socotra population to a separate taxon. Hampson (1903), Rebel (1907) and Hacker (1999) mistakenly attributed *Azygophleps inclusa* (Walker, 1856) (Fig. 15) to Socotra Island.

**Male genitalia** (Fig. 25). Oman’s *Azygophleps larseni* male paratypus specimen’s genitalia illustrated showing strong aedeagus, apically three times wider than proximally and simple, rounded, short vesica.

**Female genitalia** (Fig. 29). Papilla analis stretched, rounded apically; apophyses posteriores more than twice longer than apophyses anteriores which are forked at basal part; ductus short, wide, sclerotised basally; corpus sac-shaped, with a small star-like signum; bulla sclerotised, located on the median part of bursa.

#### Bionomics and distribution.

 This species is distributed in Iraq, Iran, Oman and mainland Yemen. Two specimens were caught in Socotra Island, ♀ (collecting date: Yemen, 500 m, Socotra isl., Ayhft riv. valley, 25 November 2008, Saldaitiene & Saldaitis leg.). *Azygophleps larseni* in Socotra is a very rare and local species. The Ayhft valley is a unique place in Socotra, with 80% of all vegetation found in Socotra Island. This valley is constantly fed by fresh water from the Haghier Mountains and its slopes are densely covered by tropical-type evergreen trees and shrubs: *Dracaena cinnabari*, *Rhus rhyrsiflora*, *Euryops arabicus*, *Buxus pedicillata*, *Gnidia socotrana*, *Cocculus balourii* and many other plants(Miller and Cope 1996).

**Figures 30, 31.**
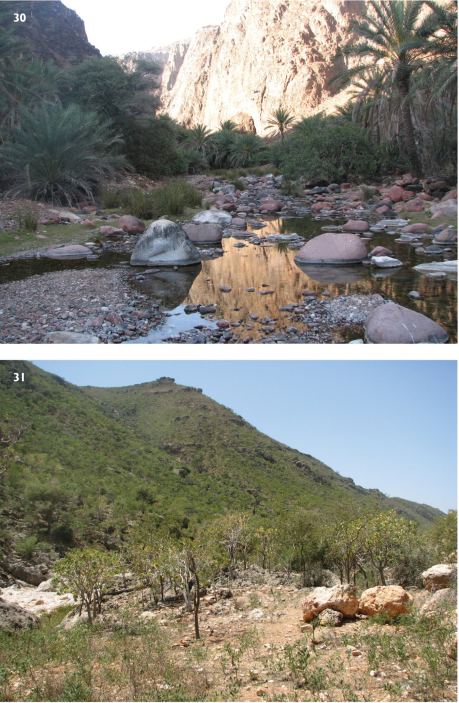
*Mormogystia* sp. and *Meharia* sp., biotopes. **30** Central part of Socotra Island, Diksam Valley. Type locality of *Mormogystia brandstetteri* sp. n. **31** Northeast Socotra Island, Wadi Difarroha Valley. Type locality of *Meharia hackeri* sp. n.

## Checklist of species

### Genus Mormogystia

*Mormogystia reibellii* (Oberthür, 1876), *Hypopta reibellii* Oberthür, 1876, Et. Ent. 1: 40, pl. 4: fig. 1. LT: Biskra [Algeria]. Distribution: North part of Saudi Arabia, North Oman, UAE, Israel, Egypt, Algeria, Libya, Tunisia, Mauritania, Niger, Chad.

= *Hypopta mussolinii* Turati, 1927, Atti Soc. Ital. Scienze Naturali 66: 322, fig. 5. LT: Giarabub [NE Libya].

= *Hypopta cognata* Krüger, 1939, Ann. Mus. Libico Storia Nat. V. 1: 331–332, Tav. 13: fig. 13–14. LT: Beni Ulid [Libya].

=*Hypopta reibelli* – Wiltshire, 1980b, Jour. Oman Stud. Special report 2: 189; An incorrect subsequent spelling of *reibellii* Oberthür, 1876.

*Mormogystia proleuca* (Hampson in Walsingham et Hampson, 1896), **stat. n.**, *Eremocossus proleuca* Hampson in Walsingham et Hampson, 1896, Proc. Zool. Soc. London: 276, pl. 10: 24. LT: Aden, Yerbury [South Yemen]. Distributuion: Southern Saudi Arabia (Asir Mountains), South Oman (Dhofar), Yemen.

*Mormogystia equatorialis* (Le Cerf, 1933), *Hypopta reibeli* (sic!) Obt. ssp. *equatorialis* Le Cerf, 1933, Bull. Soc. Entomol. France: 158. LT: Lokitang, dans les monts Lubur, au Nord du lac Rodolphe [Lokitaung, Lake Turkana, N Kenya]. Distribution: N Kenya.

*Mormogystia brandstetteri* Saldaitis, Ivinskis & Yakovlev sp. n.

### Genus Meharia

*Meharia philbyi* Bradley, 1952, Entomologist, LXXXXV (1074): 241–242: LT: Arabia, Kashabiya [Saudi Arabia]. Distribution: Saudi Arabia, Yemen, Oman.

*Meharia acuta* Wiltshire, 1982, Fauna Saudi Arab., 4:276, pl. 1: fig. 3, 3a. LT: wadi Hanaka [Saudi Arabia]. Distribution: Saudi Arabia, Oman, Yemen.

*Meharia hackeri* Saldaitis, Ivinskis & Yakovlevsp. n.

*Meharia tanganyikae* Bradley, 1952, Entomologist, LXXXXV (1074): 242–244. LT: Tanganyika, Ngaruka. Distribution: E Africa.

*Meharia semilactea* (Warren et Rothschild, 1905), Novit. zool., 12: 32, pl. 4 (12). LT: Nakheila, R. Atbara [NW Sudan]. Distribution: Israel, Jordan, Saudi Arabia, Oman, UAE, Yemen, Egypt (Sinai peninsula), N Sudan, Morocco, Mauritania.

*Meharia yakovlevi* Saldaitis & Ivinskis, 2010a, Esperiana 15: 379. LT: hills near Hadibu, Socotra Island [Yemen]. N [North]. Distribution: Yemen (Socotra Isl.).

*Meharia incurvariella incurvariella* Chrétien, 1915, Ann. Soc. Ent. Fr., 1915: 368. LT: Biskra [Algeria]. Distribution: Algeria, Morocco.

= *Blalia vittata* Rungs, [1943], 1942, Bull. Soc. Sc. Maroc. 22 (1942): 174, pl. 1: fig. 17. LT: Maroc, Saharien, Od Khiruf [Morocco].

*Meharia incurvariella persica* (Wiltshire, 1946); *Blalia vittata persica* Wiltshire, 1946a, Proc. R. Ent. Soc. London, Ser. B, 15: 120. LT: Shiraz [Fars, SW Iran]. Distribution: Iran, Afghanistan, Pakistan.

*Meharia tancredii* Sutton, 1963, Ann. Mag. Nat. Hist. 6 (13): 365–366, fig. 1–2, 6. LT: Meyan Kaleh peninsula, N Iran. Distribution: N Iran.

*Meharia scythica* D. Komarov et Zolotuhin, 2005. Nota lepid. 28 (1): 52–53, fig. 1–4. LT: [Russia] Astrakhan Prov., Akhtuba Distr., passing-track Martovsky, outsk. Bolshoe Bogdo Mt. Distribution: Russia, Volgograd and Astrakhan regions.

*Meharia fischeri* Yakovlev & Saldaitis, 2008b, Eversmannia 15–16: 49. LT: Marokko [Morocco], Jbel Bani, 3 km S Tiggane, 18 km SW Tata. Distribution: Morocco.

*Meharia avicenna* Yakovlev, 2011, Neue Entomologische Nachrichten 66: 1–129. LT: Iran, Hashtijan, 90 km S Gom. Distribution: Iran.

### Genus Aethalopteryx

*Aethalopteryx atrireta* (Hampson, 1910), *Phragmatoecia atrireta* Hampson, 1910a, Ann. Mag. Nat. Hist. 8 (6): 129; LT: Bechuanaland, Lake N’gami [Botswana]. Distribution: Botswana, S Africa.

*Aethalopteryx obscurascens* (Gaede, 1930), *Xyleutes obscurascens* Gaede, 1930, Gross-Schmett. Erde, 14: 547, Taf. 79h; LT: Maraquo, Centr. Abyss. [Central Ethiopia]. Distribution: Ethiopia.

*Aethalopteryx obsolete* (Gaede, 1930), *Xyleutes obscurascens obsolete* Gaede, 1930, Gross-Schmett. Erde, 14: 547, Taf. 79g; LT: White Nile [Central Sudan]. Distribution: Sudan, Tanzania, Swaziland.

*Aethalopteryx steniptera* (Hampson in Poulton 1916), *Duomitus steniptera* Hampson in Poulton 1916, Proc. Zoll. Soc. London: 166, pl. 2: fig. 31; LT: Somaliland, Mandera, 47 miles SW of Berbera [Somalia]. Distribution: Somalia.

*Aethalopteryx pindarus* (Fawcett, 1916), *Duomitus pindarus* Fawcett, 1916: 733; LT: Kenya, Kedai. Distribution: Kenya, Uganda, S Africa.

*Aethalopteryx wiltshirei* Yakovlev, 2009, Euroasian Entomol. J; LT: Saudi Arabia, Azir, Al Foqa, Olea-Dodonea Zone. Distribution: Saudi Arabia.

*Aethalopteryx simillima* (Hampson in Poulton 1916), *Duomitus simillima* Hampson in Poulton 1916, Proc. Zoll. Soc. London: 166, pl. 2: fig. 32; LT: Somalia, 47 miles SW of Berbera. Distribution: Somalia, Ethiopia.

*Aethalopteryx grandiplaga* (Gaede, 1930), *Xyleutes grandiplaga* Gaede, 1930: 547; LT: Chad, Oubangui, Chari, Bangui [Central African Rep.]. Distribution: Central African Rep., Congo.

*Aethalopteryx tristis* (Gaede, 1915), *Hyleutes tristis* Gaede, 1915, D. Ent. Ztschr. Iris, 28: 147–148.LT: Nama-Land [Namibia]. Distribution: Namibia, Kenya, S Africa.

*Aethalopteryx mesosticta* (Hampson in Poulton 1916), *Duomitus mesosticta* Hampson in Poulton 1916, Proc. Zool. Soc. London: 165, pl. 2, fig. 20; LT: Somalia, Mandera. Distribution: Somalia.

*Aethalopteryx diksami* Yakovlev & Saldaitis, 2010, Esperiana, Memoir 5: 333–337; LT: C Socotra [Central Socotra] isld., Top of Diksam valley. Distribution: Socotra Island, Yemen.

*Aethalopteryx squameus* (Distant, 1902), *Duomitus squameus* Distant, 1902, Entomologist, 35: 213; LT: Transvaal, Pretoria (S Africa). Distribution: South Africa, Botswana, Mozambique, Malawi, Ghana, Angola, Tanzania.

= *Azygophleps atriplaga* Le Cerf, 1919b, Bull. Mus. Nat. Hist. Nat. 25: 30; LT: Rivière Kuando, frontière Sud-Est Angola-Rhodesia [Kwando Riv., W Angola].

*Aethalopteryx dictyotephra* (Clench, 1959), *Kyleutes* (sic!) *dictyotephra* Clench, 1959, Veröff. zool. St. Samml. Münch. 6: 13–14, pl. II: fig. 6–7; LT: SW Africa, Okahandja [Namibia]. Distribution: SW Africa.

*Aethalopteryx nilotica* Yakovlev, 2011, Neue Entomologische Nachrichten 66: 1–129. LT: Sudan, Blue Nile Prov., Wadi Medani. Distribution: Sudan.

*Aethalopteryx anikini* Yakovlev, 2011, Neue Entomologische Nachrichten 66: 1–129. LT: S Africa, Free State, 15 km S Bloemhof, Sandveld N.R., S 27°43'55", E 25°45'06". Distribution: S Africa.

*Aethalopteryx forsteri* (Clench, 1959), *Xyleutes forsteri* Clench, 1959, Veröff. zool. St. Samml. Münch. 6: 14–15, pl. II: fig. 8–9; LT: SW Africa, Okahandja [Namibia]. Distribution: SW Africa.

*Aethalopteryx gyldenstolpei* (Aurivillius, 1925), *Xyleutes gyldenstolpei* Aurivillius, 1925, Ark. Zoology, 17A (32): 20; LT: Ituri [Congo, Ituri prov.]. Distribution: Congo.

*Aethalopteryx masai* Yakovlev, 2011, Neue Entomologische Nachrichten 66: 1–129. LT: Kenya, Kibwezi; Distribution: Kenya.

*Aethalopteryx elf* Yakovlev, 2011, Neue Entomologische Nachrichten 66: 1–129. LT: Somalia m., Kisimayo. Distribution: Somalia.

*Aethalopteryx politzari* Yakovlev, 2011, Neue Entomologische Nachrichten 66: 1–129. LT: Somalia m., Caanole Fluss. Distribution: Somalia, Tanzania, Kenya.

*Aethalopteryx gazelle* Yakovlev, 2011, Neue Entomologische Nachrichten 66: 1–129. LT: Kenya, South Coast, Marenche forest. Distribution: Kenya.

*Aethalopteryx rudloffi* Yakovlev, 2011, Neue Entomologische Nachrichten 66: 1–129. LT: Swaziland, Ndzevane area, Matala near Nsogo, 240 m, Akazien, Agaven Buscland, S 26°58'; E 031°58'. Distribution: Swaziland.

*Aethalopteryx kisangani* Yakovlev, 2011, Neue Entomologische Nachrichten 66: 1–129. LT: Rep. Congo (Zaire), 17 km N Kisangani, Masako Field Stat., 388 m, N 00°36'; E 25°15', 02–08.02.2008. Distribution: Zaire.

*Aethalopteryx sulaki* Yakovlev, 2011, Neue Entomologische Nachrichten 66: 1–129. LT: Kenya, Eastern Province, Umg. Meru, 2 km NE Isiolo, S 00°21.623; E 37°36.231. Distribution: Kenya.

### Genus Azygophleps

*Azygophleps liturata* (Aurivillius, 1879), *Zeuzera liturata* Aurivillius, 1879, Öfversigt af Kongl. Vetenskaps-Akademiens 7: 48–49 LT: Damara [Namibia]. Distribution: Namibia, Botswana, S Africa (Gründberg, 1910; Vári et al., 2002).

= *Zeuzera aurivillii* Kirby, 1892, Cat. Lep. Het. **1**: 872; Replacement name for *Zeuzera liturata* Aurivillius, 1879.

*Azygophleps leopardina* Distant, 1902, Entomologist 35: 213–214; LT: Transvaal, Pretoria. Distribution: S Africa, Zambia, Namibia, Kenya.

= *Azygophleps borchmanni* Grünberg, 1910, Denkschriften Med.-Naturwiss. Ges. Jena. Vierter Bd.: 140; LT: Rietfontein [E Namibia].

= *Azygophleps leopardinae* – Dalla-Torre, 1923, Lep. Cat.: 43; An incorrect subsequent spelling of Azygophleps leopardina Distant, 1902.

*Azygophleps nubilosa* Hampson, 1910; 1910a, Ann. Mag. Nat. Hist. 8 (6): 129. LT: Uganda. Distribution: Uganda, Tanzania, S Africa.

*Azygophleps atrifasciata* Hampson, 1910; 1910b, Proc. Zool. Soc. London: 481; LT: NE Rhodesia, Kalungwisi distr., High Plateau [Zambia]. Distribution: Zimbabwe, Zambia, Uganda, Kenya, Angola, Malawi, S Africa.

*Azygophleps regia* (Staudinger, 1891), *Zeuzera* (?) *regia* Staudinger, 1891, Dtsch. Entomol. Ztschr. Iris **4**: 253; LT: Hadjin [Turkey]. Distribution: Turkey, Pakistan, Iran, Iraq.

= *Zeuzera regina* – Wiltshire, 1957, Lep. Iraq: 146; An incorrect subsequent spelling of regia Staudinger, 1891.

*Azygophleps afghanistanensis* (Daniel, 1964), *Zeuzera regia afghanistanensis* Daniel, 1964, Opuscula Zool. **77**: 6; LT: O-Afghanistan, Sarobi, Gulbahar [E Afghanistan]. Distribution: Afghanistan.

*Azygophleps albofasciata* (Moore, 1879), *Zenzera* (sic!) *albofasciata* Moore, 1879a, Descr. of new ind. lep. ins. from the coll. of the late Mr. W.S. Atkinson, M.A., F.L.S. & C., director of the Public Instruction, Bengal: 87; LT: Darjiling [India]. Distribution: India, Pakistan.

*Azygophleps confucianus* Yakovlev, 2006; 2006b, Tinea 19(3): 205–207, figs, 18–19, 54; LT: China, SE Tibet, Markam; Distribution: China (SE Tibet, NW Sichuan, Yunnan, Guizhou, Qinghai).

*Azygophleps inclusa* (Walker, 1856), *Zeuzera inclusa* Walker, 1856, List. Spec. Lepid. Ins. Brit. Mus. **7**: 1534; LT: Port Natal [Durban, South Africa]. Distribution: Kenya, Tanzania, Zambia, Angola, Malawi, Mosambique, Botswana, South Africa, Lesotho, Uganda, Congo, Ghana, Sierra Leone, Guinea, Republic of Côte d’Ivoire.

= *Zeuzera petax* Wallengren, 1860, Wien. Entomol. Monatshcr 4 (2): 43; LT: Caffraria orientali [S Africa].

*Azygophleps larseni* Yakovlev & Saldaitis, 2011, Neue Entomologische Nachrichten 66: 1–129. LT: S. [South] Oman, Dhofar, Rakyut. Distribution: Iraq, Iran, Oman, Yemen, Socotra island.

*Azygophleps kovtunovitchi* Yakovlev, 2011, Neue Entomologische Nachrichten 66: 1–129. LT: Lesotho, 45 km Mokhothand. Distribution: Lesotho.

*Azygophleps sheikh* Yakovlev & Saldaitis, 2011, Neue Entomologische Nachrichten 66: 1–129. LT: W Saudi Arabia, N-Asir, 40 km W Taif, Distribution: Saudi Arabia, Yemen.

*Azygophleps sponda* (Wallengren, 1875), *Zeuzera sponda* Walengren, 1875, Öfver. Kongl. Vetenskaps-Akad. Förh. 32 (1): 96; LT: Transvaalia [S Africa, Transvaal]. Distribution: S Africa.

*Azygophleps cooksoni* Pinhey, 1968; 1968, Ann. Transvaal Mus. 25 (9): 156, pl. 13: fig. 2; LT: Muden, Natal. Distribution: Southern Africa (Natal prov.).

*Azygophleps melanophele* Hampson, 1910; 1910a, Ann. Mag. Nat. Hist. 8 (6): 130; LT: S Nigeria, Sapele [Kenya]. Distribution: Central Africa.

*Azygophleps ganzelkozikmundi* Yakovlev, 2009, Euroasian Entomol. J. 8 (3): 359–360; LT: Uele, Paulis [Congo]. Distribution: Camerun, Congo.

*Azygophleps asylas* (Cramer, 1779), *Phalaena asylas* Cramer, 1779, De uitlandsche kapellen voorkomende in de drie waereld-deelen Asia, Africa en America, by een verzameld en beschreeven: 61–62, pl. CXXXVII (C); LT: Cape [S Africa]. Distribution: Central to Southern Africa.

= *Zeuzera strigulosa* Walker, 1856, List Spec. Lep. Ins. Brit. Museum **7**: 1534;LT: Cape [S Africa].

= *Zeuzera canadensis* Herrich-Schäffer, [1854], Sammlung aussereuropäscher Schmetterlinge: 58, Fig. 168; LT: Quebec (error).

*Azygophleps pusilla* (Walker, 1856), *Zeuzera pusilla* Walker, 1856, List Spec. Lep. Ins. Brit. Museum 7: 1538; LT: North India. Distribution: India.

*Azygophleps albovittata* Bethune-Baker, 1908, Ann. Mag. Nat. Hist. (8) 2: 263; LT: N Nigeria, Lokoja District; Distribution: Nigeria, Ghana, Uganda, Congo, Kenya, Guinea, Zimbabwe.

*Azygophleps pallens* (Herrich-Schäffer, [1854]), *Phragmataecia pallens* Herrich-Schäffer, [1854], Samml. aussereurop. Schmett. 1 (1), Taf. [35]: 169; LT: Guinea. Distribution: Sierra-Leone, Uganda, Nigeria, Cameroon, Kenya, Sudan.

*Azygophleps simplex* Aurivillius, 1905, Owk. f. Zool. 2 (12): 42; LT: [Nigeria]. Distribution: Nigeria.

*Azygophleps liliyae* Yakovlev, 2011, Neue Entomologische Nachrichten, 66: 1–129. LT: Tanzania, Mbulu in town, 1800 m, S 03°52'00", E 035°32'17". Distribution: Tanzania.

*Azygophleps legraini* Yakovlev & Saldaitis, 2011, Neue Entomologische Nachrichten, 66: 1–129. LT: Cameroon, Adamaoua, nr. Ngaoundéré, Ngaoundaba; Distribution: Cameroon.

*Azygophleps godswindow* Yakovlev & Saldaitis, 2011, Neue Entomologische Nachrichten, 66: 1–129. LT: RSA [Republic South Africa], Mpumalanga, nr. Graskop, 1750 m, God’s Window Rd. Distribution: S Africa.

*Azygophleps otello* Yakovlev, 2011, Neue Entomologische Nachrichten, 66: 1–129. LT: Mauritania, Boghe. Distribution: Mauritania.

*Azygophleps equatorialis* Yakovlev, 2011, Neue Entomologische Nachrichten, 66: 1–129. LT: ♂, Congo, Odzala NP, 0,23N; 14,50E. Distribution: Congo.

*Azygophleps scalaris* (Fabricius, 1775), *Phalaena* (*Hepialus*) *scalaris* Fabricius, 1775, Syst, Ent.: 590; LT: China; Distribution: Pakistan, India, China, Sri-Lanka, Maynmar, Thailand, Cambodia, Bangladesh, Mauritania, Somali, Senegal, Republic of Côte d’Ivoire, Ghana, Nigeria, Congo, Kenya, Angola, Namibia, Tanzania, Sudan.

= *Zeuzera bivittata* Walker, 1865, List Lep. Het. Brit. Mus. 32 (suppl. 2): 586–587; LT: North Hindostan.

*Azygophleps aburae* (Plötz, 1880), *Zeuzera aburae* Plötz, 1880, Ent. Zeit. Stetting: 77; LT: Bei Aburi [Ghana]. Distribution: Zimbabwe, Kenya, Ghana, Cameroon, Sudan.

*Azygophleps boisduvalii* (Herrich-Schäffer, 1854), *Zeuzera boisduvalii* Herrich-Schäffer, 1854, Samml. aussereurop. Schmett., 1 (1): 58, Taf. 35: 167; LT: Gatam (Sierra Leone). Distribution: Africa (Guinea, Sierra Leone, Ghana, Cameroon, Nigeria, Sudan, Ethiopia, Kenya, Uganda, Congo, Zambia, Zimbabwe, Senegal, Malawi, Republic of Côte d’Ivoire).

**Table 1.** Voucher and GenBank numbers for barcoded individuals (deposited in NRCV).

*Mormogystia proleuca*	QUNOD300-10	HQ970475
*Mormogystia proleuca*	QUNOD301-10	HQ970476
*Mormogystia proleuca*	QUNOD302-10	HQ970477
*Mormogystia proleuca*	QUNOD303-10	HQ970478
*Mormogystia reibellii*	QUNOD304-10	HQ970479
*Mormogystia reibellii*	QUNOD305-10	HQ970480
*Mormogystia reibellii*	QUNOD307-10	HQ970482
*Meharia hackeri*	QUNOD309-10	HQ970483
*Meharia hackeri*	QUNOD310-10	HQ970484
*Meharia hackeri*	QUNOD311-10	HQ970485
*Meharia acuta*	QUNOD312-10	HQ970486
*Mormogystia brandstetteri*	QUNOD336-10	HQ970510
*Mormogystia brandstetteri*	QUNOD337-10	HQ970511
*Mormogystia brandstetteri*	QUNOD338-10	HQ970512
*Mormogystia brandstetteri*	QUNOD339-10	HQ970513

## Supplementary Material

XML Treatment for Mormogystia

XML Treatment for Mormogystia brandstetteri

XML Treatment for Meharia

XML Treatment for Meharia hackeri

XML Treatment for Meharia yakovlevi

XML Treatment for Aethalopteryx

XML Treatment for Aethalopteryx diksami

XML Treatment for Azygophleps

XML Treatment for Azygophleps larseni
